# Fish oil-based lipid emulsion in the treatment of parenteral nutrition-associated cholestasis

**DOI:** 10.1186/s13052-018-0539-0

**Published:** 2018-08-23

**Authors:** Simonetta Costa, Rossella Iannotta, Luca Maggio, Giovanni Barone, Francesca Serrao, Giovanni Vento

**Affiliations:** Department of Woman and Child Health, Obstetric and Neonatology Area, Fondazione Policlinico Universitario A. Gemelli IRCCS, Largo A. Gemelli 8, 00168 Rome, Italy

**Keywords:** Fish oil-based lipid emulsion, Parenteral nutrition-associated cholestasis, Preterm infants

## Abstract

**Background:**

Parenteral nutrition-associated cholestasis (PNAC) is a serious complication in preterm infants receiving prolonged parenteral nutrition. Soybean lipid emulsion (SLE) seems to have a role in its pathogenesis, whereas fish oil-based emulsion (FOLE) seems to be able to reverse cholestasis. This study aimed to evaluate the effectiveness of a FOLE in reversing PNAC.

**Methods:**

The effectiveness in reversing PNAC was evaluated in prospective cohort study of very preterm infants when compared to historical controls: twenty-six infants (27.0 ± 2.6 weeks GA; 724 ± 204 g) who developed cholestasis while receiving SLE were shifted to receive FOLE and were compared with 30 infants (27.3 ± 2.5 weeks GA¸ 838 ± 277 g) who continued to receive SLE at diagnosis of cholestasis.

**Results:**

Time to reversal of cholestasis was the same in the two study groups (45 ± 21 vs 43 ± 32 days).

**Conclusions:**

FOLE does not seem to be superior to SLE in reversing cholestasis. Considering that definitive data on the actual efficacy of FOLE to reverse PNAC are lacking, larger randomized trials are required, mainly to asses if FOLE may have a role in PNAC prevention rather than PNAC treatment.

## Background

Parenteral nutrition (PN) is a life-saving therapy, providing nutrients to preterm neonates who are unable to tolerate enteral nutrition. Its long-term use is associated with serious complications, such as PN-associated cholestasis (PNAC). The incidence of PNAC is variable, from 12 to 85%, being inversely proportional to gestational age [1].

Risk factors for PNAC include low birth weight, prematurity, prolonged PN use, lack of enteral intake, enzyme deficiencies, genetic causes, anatomic factors, susceptibility to cholestatic injury, and factors relevant to the PN itself. A further risk factor is the occurrence of severe infections, due to the requirement for central line for infusion of PN, and bacterial overgrowth caused by enteral starvation and immature immune function [2].

The most effective treatment for PNAC is increasing enteral energy intake while weaning off PN but, in about 3–15% of patients, end-stage liver disease develops and liver/small bowel transplantation remains the only treatment option. Mortality rate in cholestatic infants with end stage liver disease almost reaches 100% within one year from the diagnosis, unless PN can be discontinued or liver/small bowel transplantation can be performed [3].

Intravenous lipid emulsions seem to have a significant role in the pathogenesis of PNAC [4].Several reports and clinical studies have found reversal of PNAC when replacing standard soy-based parenteral lipid emulsion (SLE) with fish oil-based lipid emulsion (FOLE) [5–12].

Soybean-based emulsions may be involved in the aetiology of PNAC for several reasons. They are rich in ω-6 fatty acids, which have pro-inflammatory metabolites that can contribute to impaired immunologic function. Moreover soybean-derived lipid emulsions contain phytosterols, a group of plant steroid compounds that have been shown to decrease bile flow in animal models and that are believed to be hepatotoxic. On the contrary, fish-oil-based emulsions, rich in ω-3 fatty acid metabolites, including docosahexaenoic and eicosapentaenoic acids, can enhance anti-inflammatory pathways [13–15].

In this study we assessed the efficacy of a FOLE to reverse PNAC in a population of very low birth weight preterm infants who developed cholestasis while receiving SLE.

## Methods

This study was conducted at A. Gemelli Hospital in NICU. In January 2010 we started to use FOLE in infants with PNAC who met the following criteria: birth weight < 1500 g, PN with SLE for more than 14 days, and predicted PN infusion > 30 days.

All infants with PNAC were switched from SLE to FOLE at the time of diagnosis. These infants were prospectively studied (FOLE group) and compared with an historical cohort of infants, admitted to our unit between 2005 and 2009 (SLE group). Infants in the SLE group continued to receive SLE at the diagnosis of cholestasis. PNAC was defined as a concentration of direct bilirubin ≥2 mg/dL on 2 consecutive measurements in a patient receiving NP for ≥2 weeks, without other liver diseases (i.e., cystic fibrosis, inborn metabolic errors, and hepatitis) [1]. The institutional review boards approved the study, and written informed consent was obtained from the parents of all subjects enrolled in the FOLE group.

A historical cohort of infants, admitted to our unit between 2005 and 2009 with PNAC was used as comparison group. At that time SLE was continued at the diagnosis of cholestasis (SLE group).

In the FOLE group, at the moment of diagnosis of PNAC, treatment with fish-oil–based emulsion (Omegaven, Fresenius Kabi, Italy) was started. Patients received fish-oil emulsions at 1.5 g/Kg/die and soybean-based emulsion (Intralipid, Fresenius Kabi, Italy) at 0.5 g/Kg/die. A total amount of 2 g/kg/die of lipids was administered. A minimal amount of soybean lipid emulsion was maintained to avoid the possibility of essential fatty acid deficiency syndrome.

SLE group continued to receive soybean emulsion at a reduced dose of 2 g/Kg/die from a median dose of 3.5 g/Kg/die received previously.

The primary study outcome was time to reversal of cholestasis, defined as the time needed to obtain two consecutive serum direct bilirubin < 2 mg/dL, starting from the day when therapeutic intervention was applied. Laboratory tests, including serum total and direct bilirubin, lipid profile and blood clotting tests, were prospectively measured at approximately weekly intervals. The same data were available for the SLE group.

For each infant, maximum direct bilirubin level, age at the onset and the age at the resolution of PNAC were recorded. The presence of abdominal pathology and sepsis, duration of PN, length of hospital stay and mortality were also considered.

Statistical significance of baseline differences between the 2 groups was assessed via *t* tests when reporting means (or Wilcoxon tests when reporting medians) and *x*^2^ tests when reporting proportions (or Fisher’s exact tests when warranted). A *p* value below 0.05 was considered statistically significant.

## Results

At baseline, there were no significant differences in characteristics of the two groups (Table 1).

**Table 1:** Baseline characteristics of infants in the FOLE and SLE Cohorts

	FOLE group 26	SLE group 30	p
Gestational age (weeks)	27.0 ± 2.6	27.3 ± 2.5	0.629
Birth weight (g)	724 ± 204	838 ± 277	0.090
Male (%)	13 (50)	17 (56)	0.788
SGA (%)	9 (35)	8 (27)	0.570
NEC (%)	9 (35)	6 (20)	0.243
Sepsis (n)	1.5 ± 0.7	1.6 ± 0.5	0.619
PN duration (days)	71.7 ± 31.1	56.8 ± 28.2	0.065
Lenght of stay (days)	128 ± 66	127 ± 64	0.965
Mortality (%)	3 (11)	5 (16)	0.715

Data are presented as mean ± SD or number (percentage)

All the patient who developed necrotizing enterocolitis (NEC) in both groups underwent surgery to perform ileostomy. All the NEC were NEC stage >2a, according to the modified Bell criteria [16].

The time to reversal PNAC was the same in the two study groups. Similar were also the time of diagnosis and the severity of the PNAC (Table 2). Neither alteration of the lipid profile (hypertriglyceridemia and hypercholesterolemia), or abnormal coagulation was found in both study groups (data not shown).

**Table 2:** Primary outcome and data related to cholestasis

	FOLE group 26	SLE group 30	p
Days to obtain resolution of PNAC	45 ± 21	43 ± 32	0.718
Resolution of PNAC (age in days)	76 ± 33	75 ± 27	0.917
Onset of PNAC (age in days)	35 ± 14	29 ± 18	0.214
Maximum direct bilirubin level (mg/dL)	8.2 ± 4.0	7.4 ± 3.7	0.461

Data are presented as mean ± SD

## Discussion

Our results suggest that fish-oil–based lipid emulsions, at dosage of 1.5 g/Kg/die, have the same efficacy to reverse PNAC of standard soybean lipid emulsions, at dosage of 2 g/Kg/die.

FOLE group and SLE group are comparable. In fact there is not any statistical differences in the variables that could have affected PNAC other than lipid emulsion. A) There was no difference in the number of sepsis and considering the almost perfect overlap in hospital stay, we can reasonably assume that the number of infection per day per patient is also similar between the two groups. B) There was no difference in the length of parenteral nutrition which might be considered a proxy about the progression and quantity of enteral feeding. C) None of the patients was ever treated with Ursodeoxycholic acid.

These results are in contrast with those reported in the in the last decade literature, that have shown a tendency to resolution of the PNAC in patients receiving fish-oil based lipid emulsions in comparison with patients who continued to receive soybean lipid emulsions [5–12]. This disagreement may be due to the fact that in the historical cohort of infants, the management of PNAC included the reduction of total lipid intake to 2 g/Kg/die and this intervention represents itself a measure for the resolution of the PNAC [17]. Moreover, the small number of infants included in our study could have failed to find a difference in the primary outcome. However if we consider the number of patient in each group and the standard deviation in the number of days to obtain the resolution of PNAC we would have been able to detect at least 10 days difference in the main outcome with alpha = 0.05 and 1-beta = 0.80.

Our results agree, indeed, with those of Lam et al., who found in their prospective, double-blind, randomized controlled trial, no difference in reversal of PNAC at 4 months between FOLE and SLE groups, although they noticed a significantly higher increase of plasma-conjugated bilirubin and alanine aminotransferase in the SLE group, suggesting a slower progression of PNAC using fish-oil lipid emulsion [18].

A recent systematic review investigating effects of different lipid emulsions on several items suggests that there are limited differences in relevant laboratory or clinical outcomes in paediatric patients ranging from preterm infants to children < 18 years of age [19].

We are aware that using a historical cohort for comparison purposes may result in bias, but variables such as gestational age, incidence of sepsis and necrotizing enterocolitis, duration of parenteral nutrition, that may influence the occurrence and the severity of PNAC, were similar in two groups. Furthermore, our findings do not discourage the growing interest in possible benefits of FOLE, but they highlight that more information is needed in regards to their real efficacy in the treatment of PNAC. Moreover, our finding underlines the fact that the use of lipid restriction and lipid replacement in the treatment of PNAC remains controversial and requires further study [20].

## Conclusions

Considering that fish-oil emulsions have a potential protective role towards liver, and that most of the available studies are retrospective, lacking high-quality prospective randomized controlled trials, we believe that larger randomized trials are required to asses if more than as treatment of an already established PNAC, the use of fish-oil based emulsions should be introduced in a preventive approach, in particular to those classes of newborns that, for their characteristics, appear predisposed to a longer duration of PN.

## Abbreviations

FOLE: Fish oil lipid emulsion
PN: Parenteral nutrition
PNAC: Parenteral nutrition-associated cholestasis
SLE: Soybean lipid emulsion
